# Effect of polyvinyl alcohol and carboxymethylcellulose on the technological properties of fish gelatin films

**DOI:** 10.1038/s41598-022-14258-y

**Published:** 2022-06-21

**Authors:** Gleyca de Jesus Costa Fernandes, Pedro Henrique Campelo, Jayne de Abreu Figueiredo, Hugo Junior Barbosa de Souza, Maria Regina Sarkis Peixoto Joele, Maria Irene Yoshida, Lúcia de Fátima Henriques Lourenço

**Affiliations:** 1 Animal Research Laboratory - LAPOA, Graduate Program in Food Science and Technology - PPGCTA, Federal University of Pará - UFPA, Belém, PA Brazil; 2 Department of Food Technology, Federal University of Vicosa, Av. PH Rolfs, s/n, Vicosa, MG 36570-900 Brazil; 3 Food Science Department, Federal University of Lavras, Lavras, MG 37200-000 Brazil; 4 Pará Federal Institute of Education, Science and Technology - IFPA, Castanhal, PA Brazil; 5 Chemical Department, Federal University of Minas Gerais, Belo Horizonte, MG Brazil

**Keywords:** Chemistry, Materials science

## Abstract

The objective of this work was to develop biodegradable films by mixing gelatin/carboxymethylcellulose (FG/CMC) and gelatin/polyvinyl alcohol (FG/PVOH) and to evaluate the effect of adding these polymers on the properties of fish gelatin films. The films FG/CMC and FG/PVOH were produced in the proportions 90/10, 80/20 and 70/30 and characterized their physical, chemical and functional properties. The addition of CMC and PVOH improved the mechanical strength, barrier property and water solubility of gelatin films. FG/CMC films showed greater tensile strength and greater solubility than FG/PVOH. The maximum concentration of CMC promoted the highest mechanical resistance, while the highest PVOH content produced the film with the lowest solubility. The proposed mixing systems proved to be adequate to improve the properties of fish gelatin films, with potential for application in the packaging sector.

## Introduction

New technologies and the demands of the current world, as well as the large consumption of processed foods that require packaging (primary, secondary and tertiary), have caused an increase in the generation of solid waste, which remains for hundreds and/or thousands of years in the environment, causing an environmental crisis, as well as economic and social problems^1^. Given this, there has been growing concern about the fate of plastic packaging derived from oil due to the serious environmental problems and ecological risks that can already be observed^2,3^.

Biodegradable film packages are thin layers previously formed as plastic films, which are generally produced through the casting technique or extrusion and are used to wrap foods^4^. The biodegradable films produced from biopolymers have an essential role in reducing the environmental impact caused by the disposal of non-biodegradable plastic residues^5^, once they present potential for reducing, or substituting petroleum-based plastics. Such biological-based films must offer all the necessary functions of containment, protection, preservation and information, in an economical and environmentally friendly way^6^.

As an alternative to films produced with conventional materials, many studies have been performed using biodegradable, non-toxic, biocompatible and renewable sources to develop films that do not pollute the environment^7^. Among these sources, gelatin, an animal protein resulting from the partial hydrolysis of collagen^8^, stands out as a promising raw material for developing biodegradable food packaging, due to excellent characteristics such as formation of films and light and oxygen barrier properties^9^.

Fish gelatin extracted from processing residues, such as bones and skins is a potential alternative to mammals, due to the socio-cultural and health aspects involved in obtaining bovine and porcine sources^10^. In addition, the use of waste for the development of biodegradable films has important benefits for the fishing industry and the environment^11^. Although gelatin films have appropriate characteristics for film making^12^, the hydrophilic nature of this material can limit its use^13^. Gelatin films have lower mechanical resistance and low water resistance when compared to conventional plastics, being one of the main disadvantages for its use as packaging material.

Much research is being carried out in order to improve the functional properties of these films^11^, including mixing with other polymers is an alternative to make the properties of gelatin films more efficient. The mixture of polymers, such as proteins/polysaccharides and/or synthetic/natural polymers, makes it possible to obtain composite films, resulting in materials with better properties than pure components. The physical and chemical properties of the films can be improved, enabling numerous applications, resulting in good performance in the final product^6^.

Carboxymethylcellulose (CMC) is an anionic cellulose derivative produced through an etherification reaction, by partially replacing hydroxyl groups of cellulose with carboxymethyl. The food, medicine and textile industries have great interest in this polymer due to its biodegradability, biocompatibility, availability, non-toxicity and good mechanical and barrier properties, in addition to presenting favorable characteristics to form films^14,15^.

Polyvinyl alcohol (PVOH) is a synthetic polymer that is also important in the packaging area^16^, of high polarity^17,18^, low cost, non-toxic and biodegradable^19^, being produced commercially by hydrolysis of acetate of polyvinyl^20^. This material has received great attention due to its excellent oxygen barrier properties, mechanical properties, chemical resistance, film-forming capacity, water solubility, mechanical properties and biocompatibility^21,22^. CMC and PVOH have been widely used in polymer blends to produce new polymeric materials with improved characteristics^22–25^.

Developing films from the mixture of gelatin (FG) with other polymers such as carboxymethylcellulose and/or polyvinyl alcohol is a viable alternative for the use of residues generated by the fishing industry, reducing the environmental impact, and at the same time making the properties of more functional gelatin films, increasing their potential use as biodegradable packaging. Therefore, the objective of this work was to produce biodegradable films by mixing gelatin/carboxymethylcellulose (FG/CMC) and gelatin/polyvinyl alcohol (FG/PVOH) and to evaluate the effect of adding these polymers on the properties of fish gelatin films.

## Material and methods

### Materials

Fish gelatin, the main component of the film formulations, was extracted from yellow hake skin (*Cynoscion acoupa*), donated by Indústria de Pesca, Ecomar Ltda (Vigia, PA, Brazil). Sodium chloride (NaCl) (Exodus Cientifica, Sumaré, SP, Brazil), Sodium hydroxide (NaOH) (Exodus Cientifica, Hortolândia, SP, Brazil), Acetic acid (C_2_H_4_O_2_) (Cinética, Itapevi, SP, Brazil), Sodium Salt Carboxymethylcellulose—CAS 9004-32-4 (Neon, Suzano, SP, Brazil), Polyvinyl Alcohol HD = 89.5% hydrolyzed—CAS 9002-89-5 (Exodus Cientifica, Sumaré, SP, Brazil) and Glycerol—CAS 56-81-5 (Isofar, Rio de Janeiro, RJ, Brazil) were purchased from local businesses.

### Extraction of gelatin

The extraction of fish gelatin was carried out according to the methodology described by^26^. The skins were cut into 4 cm × 4 cm, washed in running water and immersed in 0.6 M sodium chloride (NaCl)—CAS 7647-14-5 solutions for 15 min, 0.3 M sodium hydroxide (NaOH)—CAS 1310-73-2 for 15 min and lastly in acetic acid (C_2_H_4_O_2_)—CAS 1189-52-3 0.02 M for 60 min, in the proportion 1:5 (weight/volume). All stages occurred under agitation (85 rpm at 25 °C), with the skin washed in water, with 3 repetitions. Subsequently, the skins were placed in a water bath (Tecnal, Te-057, Brazil) at 60 °C for 6 h to extract the gelatin, and the material obtained was filtered through faillet fabric. Then, the obtained solution was placed in stainless steel trays for drying in an oven (Tecnal, Te-394/3, Brazil) at 50 °C for 16 h. Finally, the gelatin was vacuum packed (Fastvac Wrapping Machine, F200, Brazil) and stored at − 22 °C until the films were made.

### Preparation of films

The films were produced by casting. The films were made from mixtures of polymeric solutions FG/CMC and FG/PVOH in the proportions 90/10, 80/20 and 70/30, with a total concentration of 3% (m/v) of solution and 10% (m/m polymers) of plasticizer.

The filmogenic solutions were prepared separately, dissolving gelatin (G), carboxymethylcellulose (CMC) and polyvinyl alcohol (PVOH) in distilled water. To prepare the gelatin filmogenic solution (FG), glycerol was added as a plasticizer and heated in a water bath (Tecnal, TE-057, Brazil) at 70 °C for 15 min. The CMC and PVOH solutions were heated to 70 and 90 °C, respectively, for a period of 60 min, and then mixed with the FG solution.

The FG/CMC filmogenic solutions were placed on a shaking plate (Quimis, Q-221.1, Brasil) and the FG/PVOH were stirred in a homogenizer (Ultra Stirrer-380, Brazil), both for 30 min. Finally, they were placed on a silicone support (22 cm in diameter × 3 cm in height) and dried in an oven (Tecnal, Te-394/3, Brazil) at 30 °C for approximately 15 h. The films obtained were vacuum packed and kept at room temperature. A control film (FG) was also made without the addition of CMC and PVOH.

### Characterization of films

#### Thickness

The thickness was determined using a digital micrometer with a resolution of 0.001 mm (Insize Co., modelo IP54, São Paulo, SP, Brasil). Eight random locations were selected around each film obeying a 60 mm edge spacing.

#### Mechanical properties

To determine the tensile strength and percentage of elongation at the break of the films, texture analyzer equipment (Stable Micro Systems, model TA. XT-Plus, England) was used, according to the standard method ASTM D882-91^27^, in which the initial separation of the grips and the probe speed were of 20 mm and 1 mm s^−1^, respectively. The films were cut into pieces of 60 mm × 25 mm (length × width) and the tensile strength (TS) and the elongation percentage (% E) were calculated by Eqs. (1) and (2), respectively. The analysis was performed in triplicate.

1 $$TS= \frac{Fm}{A}$$

2 $$E= \frac{{d}_{T}}{{d}_{inicial}} \times 100$$

where TS: tensile strength (MPa); Fm: maximum force when the film breaks (N); A: cross-sectional area of the film (m^2^); E: elongation (%); d_T_: total distance at the moment of rupture (mm); d initial: initial separation distance of the claws (50 mm).

#### Water vapor permeability

The water vapor permeability (WVP) of the films was measured by the modified ASTM D882-95 method, described by^28^. The samples were sealed with silicon adhesive (Orbi Química Co., Leme, SP, Brazil) in the circular opening of a glass permeation container of 4.5 × 7.0 cm (inside diameter × height) containing 10 g of silica gel (0% 0% RH; 0 Pa of water vapor pressure at 30 °C). These containers were placed in desiccators containing distilled water at 30 °C (99% RH; 4244.9 Pa of water vapor pressure at 30 °C). They were weighed at 1 h intervals for a period of 10 h. WVP was calculated from Eq. (3) and the analysis was performed in triplicate.

3 $$WVP=\frac{W\cdot X}{A \cdot t \cdot \Delta P}$$

where WVP: water vapor permeability (gm m^−2^ S^−1^ Pa^−1^); W: weight gain from the desiccant (g); X: film thickness (m); A: surface area of the exposed biofilm (m^2^) t: incubation time (hours); ∆P: partial pressure difference (Pa).

#### Solubility

To determine solubility, the films were cut into 2 cm diameter discs and placed in an oven at 105 °C for 24 h and weighed. Subsequently, immersed in containers containing 50 ml of water and shaken in a refrigerated Shaker incubator (Lucadema, model LUCA-223) with a speed of 150 rpm for a period of 24 h at 25 °C. Finally, the samples were dried (105 °C for 24 h), to determine the dry matter not dissolved in water^29^. The analysis was performed in triplicate.

#### Color and opacity parameters

The instrumental color of the samples was determined with a portable colorimeter (model CR 400, Konica Minolta Co., Chiyoda , Tokyo, Japan), obtaining parameters of L^*^ (brightness), a^*^ (intensity of red), b^*^ (intensity of yellow) and the total color difference (ΔE^*^) was calculated according to Eq. (4), in relation to the control film (FG).

4 $${\Delta E}^{*}= \sqrt{{(\Delta L}^{*}{)}^{2}+{(\Delta a}^{*}{)}^{2}+{(\Delta b}^{*}{)}^{2}}$$

where Δ is the difference of the color parameters of the biofilm sample in relation to the white standard (L: 95.59; a: − 5.56; b: 8.16).

Opacity was analyzed using a spectrophotometer (DU 640, Bachman, USA), at a wavelength of 600 nm and absorbance was measured. The opacity of the films was calculated using Eq. (5), according to the method described by^30^.

5 $$O = {\text{Abs}}_{600} /{\text{x}}$$

where *O* = opacity; Abs_600_ = absorbance at 600 nm and x = film thickness (mm).

#### Scanning electron microscopy (SEM)

To obtain the micrographs, the samples were fixed on supports with double-sided adhesive tape, metallized with gold, at an approximate thickness of 15 nm (Emitech, model K550X, England), with a covering time of 1.5 min. The cross-sectional images of the films were obtained using a scanning electron microscope (Zeiss, model EVO-MA-10, Germany) at a constant acceleration voltage of 10 kV and magnification of 500×, with an acceleration voltage of 10 kV, electron beam current of 100 µA and a working distance of 8.5 mm.

#### Thermogravimetric analysis (TGA)

The thermogravimetric curves were obtained using a TGA-50 Shimadzu, Japan analyzer, according to^31^. The samples were submitted to a heating rate of 10 °C/min, in an atmosphere of nitrogen at 50 ml/min) in the temperature range of 25–600 °C.

#### Fourier transform infrared spectroscopy (FTIR)

FTIR analyzes were performed using an Agilent spectrometer, model Cary 630 using the Total Attenuated Reflectance technique in the range of 650–4000 cm^−1^, with 4 cm^−1^ resolution and accumulation of 32 scans.

#### X-ray diffraction (XRD)

X-ray diffraction (XRD) measurements were performed on a Siemens D5000 X-ray diffractometer with Cu-Kα radiation (*λ* = 1.78901 nm) accelerated in voltage and current of 40 kV and 40 mA, respectively. The XRD pattern was collected in the scan range 2*θ* of 5°–80° at a step size of 0.02°/min.

### Statistical analysis

The analysis results were subjected to analysis of variance (ANOVA) and Tukey test (*p* < 0.05), analyzed using the Statistica® version 7.0 program^32^.

## Results and discussion

### Thickness

**Figure 1:**
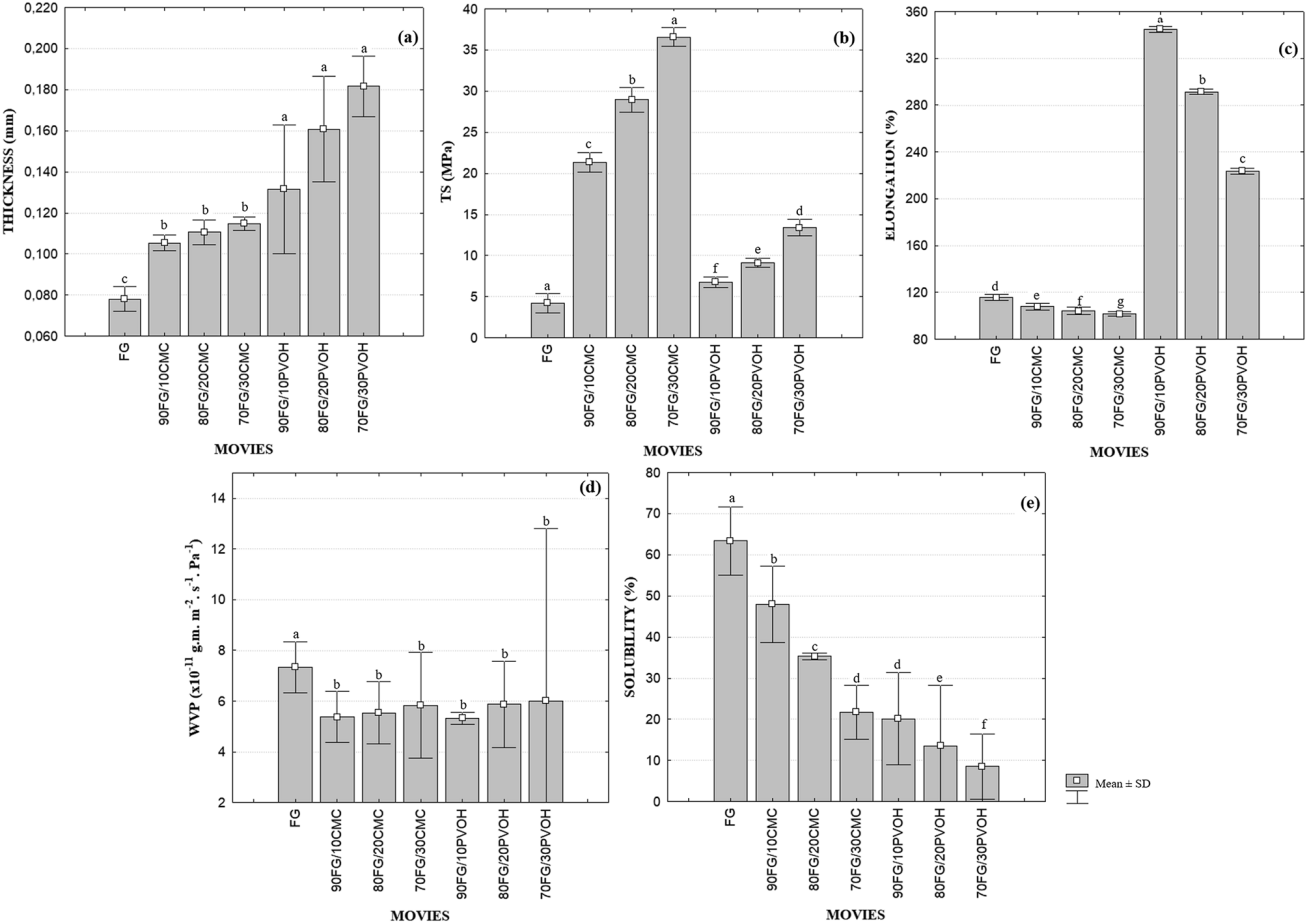
Means and standard deviations (SD) of the following properties: (**a**) Thickness; (**b**) Tensile strength (TS); (**c**) Elongation (% E); (**d**) Water vapor permeability (WVP); (**e**) Solubility of FG films, 90FG/10CMC, 80FG/20CMC; 70FG/30CMC; 90FG/10PVOH, 80FG/20PVOH, 70FG/30PVOH. Different letters represent significantly different values (*p* < 0.05).

The results showed a difference (*p* < 0.05) between the thickness values (Fig. 1a), and the films with CMC and PVOH showed greater thickness when compared to the control (FG). This can be attributed to changes in the microstructure due to interactions between polymers^33^. The results indicate that this parameter is related to the type of polymer added (CMC and PVOH), regardless of the concentration used.

### Mechanical properties

The control (FG) showed low tensile strength, which is characteristic of films made with fish gelatin, which restricts its use as food packaging^34^. The amount of CMC added to the film matrix increased (*p* < 0.05) the TS values, making the films more resistant compared to gelatin (FG), however, with reduced elongation (% E) (Fig. 1b). The maximum TS value was obtained with 30% of CMC in the mixture, in contrast this same composition revealed the lowest % E. A similar behavior to the one found in this study was observed by^35^ when assessing protein-polysaccharide films.

This behavior may be due to the interactions between gelatin and CMC, which made the film more resistant, suggesting increased structural cohesion of the films. Such characteristic is related to the ability of the polymer to form strong and numerous bonds between two polymeric chains, making their rupture more difficult when subjected to mechanical forces^36^. And the increase in structural cohesion causes a reduction in the flexibility of the film and, consequently, in the percentage of % E^37^. According to^38^, the mechanical characteristics of the mixing film depend on the intermolecular forces between the polymer chains and the molecular symmetry of each polymer.

Besides the interactions between polymers, the excellent mechanical strength of CMC gelatin films can be partially explained in terms of their semi-crystalline structure^35^, once the greater the crystallinity, the greater the resistance to traction compared to the amorphous structure, because the crystallinity reduces the degree of freedom for the mobility of the molecules^39^.

FG/PVOH films showed greater tensile strength than the control film, as it was added and/or increased the proportion of PVOH in the mixture. However, lower than TS of CMC films. A similar phenomenon has been reported by other researchers^40,41^. Regarding elongation, PVOH is a polymer capable of producing highly extensible films, as described by^42,43^.

According to^44^, the occurrence of intermolecular interactions between polymers significantly improves the tensile strength, due to the formation of inter and intramolecular hydrogen bonds. PVOH has good mechanical properties due to its flexible C–C bonds and the presence of a large number of OH groups in their chains^45^. In the presence of several hydroxyl groups, PVOH is able to act as a plasticizer, increasing the molecule free volume and mobility and, consequently, making the polymeric matrix less dense, thus improving flexibility^46^. In this study, the increase in %E of the FG/PVOH films possibly occurred because the incorporation of PVOH into the polymeric matrix reduces the number of interactions between the protein chains of the gelatin, and promotes bonds between them and other PVOH chains through hydrogen bonds^47^, as shown in the FT-IR results.

In general, the incorporation of CMC as well as PVOH in gelatin films positively influenced the mechanical properties, making the films more resistant and flexible.

### Water vapor permeability

The composite films FG/CMC and FG/PVOH showed lower permeability to water vapor in all studied concentrations (*p* < 0.05) when compared to the control (Fig. 1c). The reduction in WVP in films with CMC may be due to the strong intermolecular interaction between the chains of proteins and polysaccharides, such as hydrogen, hydrophobic and electrostatic bonds^48^, reducing the space free and intermolecular distance in the matrix, making diffusion of water molecules more difficult^49^. The highly crystalline and hydrophobic character of cellulose fibers may also have affected the film's WVP. The incorporation can produce a tortuous path, blocking the passage of water molecules through the film matrix^50^.

As for the composite FG/PVOH films, the addition of polyvinyl alcohol allowed the OH groups of this polymer to interact with the gelatin chains by intermolecular forces such as hydrogen bonds, reducing the number of hydrophilic groups. The strong interactions between the polymer chains in the films reduce the permeation of water vapor molecules through the structural matrix, reducing the WVP values^41^. In addition, the more ordered molecular structure of PVOH, as well as a greater number of OH groups, reflect an increase in the polarity and crystallinity of FG/PVOH composite films.

The concentrations of CMC and PVOH studied did not influence the WVP of the films, nor did the type of polymer affect the interaction of the films with water (*p* > 0.05). The WVP values found for the studied composite films are in accordance with the results presented previously performed with polymer mixtures^51–53^.

### Solubility

The solubility of the FG control film was the highest (Fig. 1d) and similar to the value (64%) reported by^54^ for fish skin gelatin films. The high solubility of these films is a limitation to replace the use of conventional plastics, especially for applications such as packaging, due to the hydrophilic character of the proteins (presence of polar peptides) and the relevant content of hydrophilic plasticizer (glycerol) added to give elasticity to the film^34^.

The addition of CMC and PVOH significantly reduced (*p* < 0.05) the solubility of the films. The concentrations of CMC used are adequate to provide the entanglement of the gelatin polypeptide chains with the added polymers. This interference can provide a significant blockage of the gelatin's ability to interact with water molecules^35^.

In FG/PVOH films, the reduction in solubility may be due to the formation of hydrogen bonds between gelatin and PVOH molecules, which decreased the amount of hydroxyl groups available, making it difficult to associate the polymer with water molecules^33^. The results indicated that the combination of polymers improves the integrity and water resistance of gelatin films, an important property for the use of the material as a biodegradable packaging for foods with high humidity.

### Color and opacity parameters

**Figure 2:**
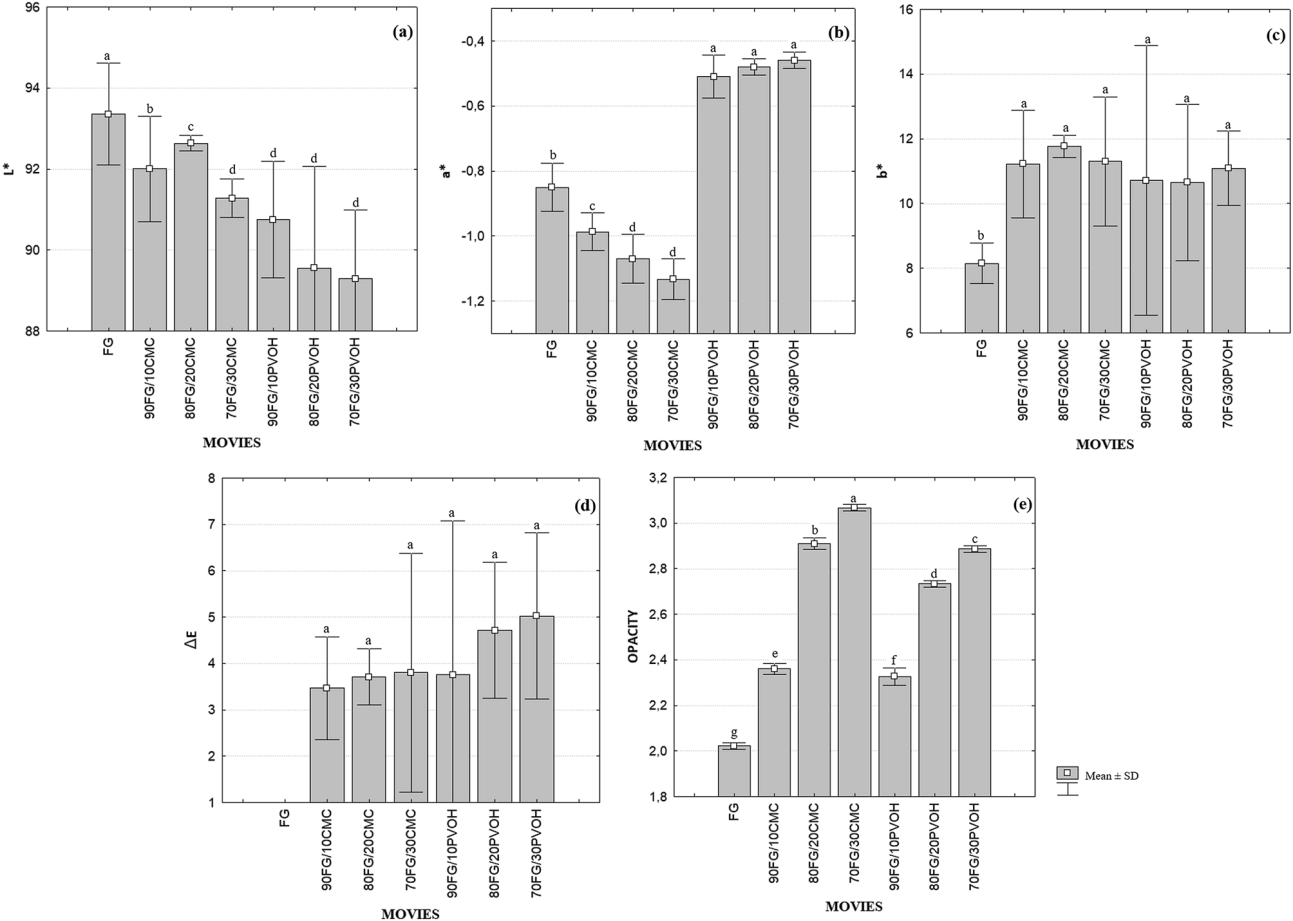
Means and standard deviations (SD) of the color parameters: (**a**) L^*^; (**b**) a^*^; (**c**) b^*^, (**d**) ΔE and (**e**) opacity of the FG, 90FG/10CMC, 80FG/20CMC; 70FG/30CMC; 90FG/10PVOH, 80FG/20PVOH, and 70FG/30PVOH films. Different letters represent significantly different values (*p* < 0.05).

Color and opacity can directly affect the appearance of food and consumer satisfaction, therefore, they are important packaging properties^55^. The color tests showed that the addition of CMC and PVOH influenced the color parameters L^*^, a^*^, b^*^ (Fig. 2a–c).

For all films analyzed, the values of a^*^ are negative, indicating the presence of a green component, while parameter b^*^ is positive with a tendency to yellow. For FG/CMC films, there was a reduction in the values of L^*^ and a^*^ and an increase in b^*^ (*p* < 0.05), when compared to the control film, that is, they reflect less light. The FG/PVOH mixture had a similar behavior regarding the values of L^*^ and b^*^, however the parameter a^*^ showed higher values. As the values of a^*^ and b^*^ were close to zero, it is considered that the films presented a slightly gray color. The total color difference values ΔE were not affected by the amount and type of polymer added.

**Figure 3:**
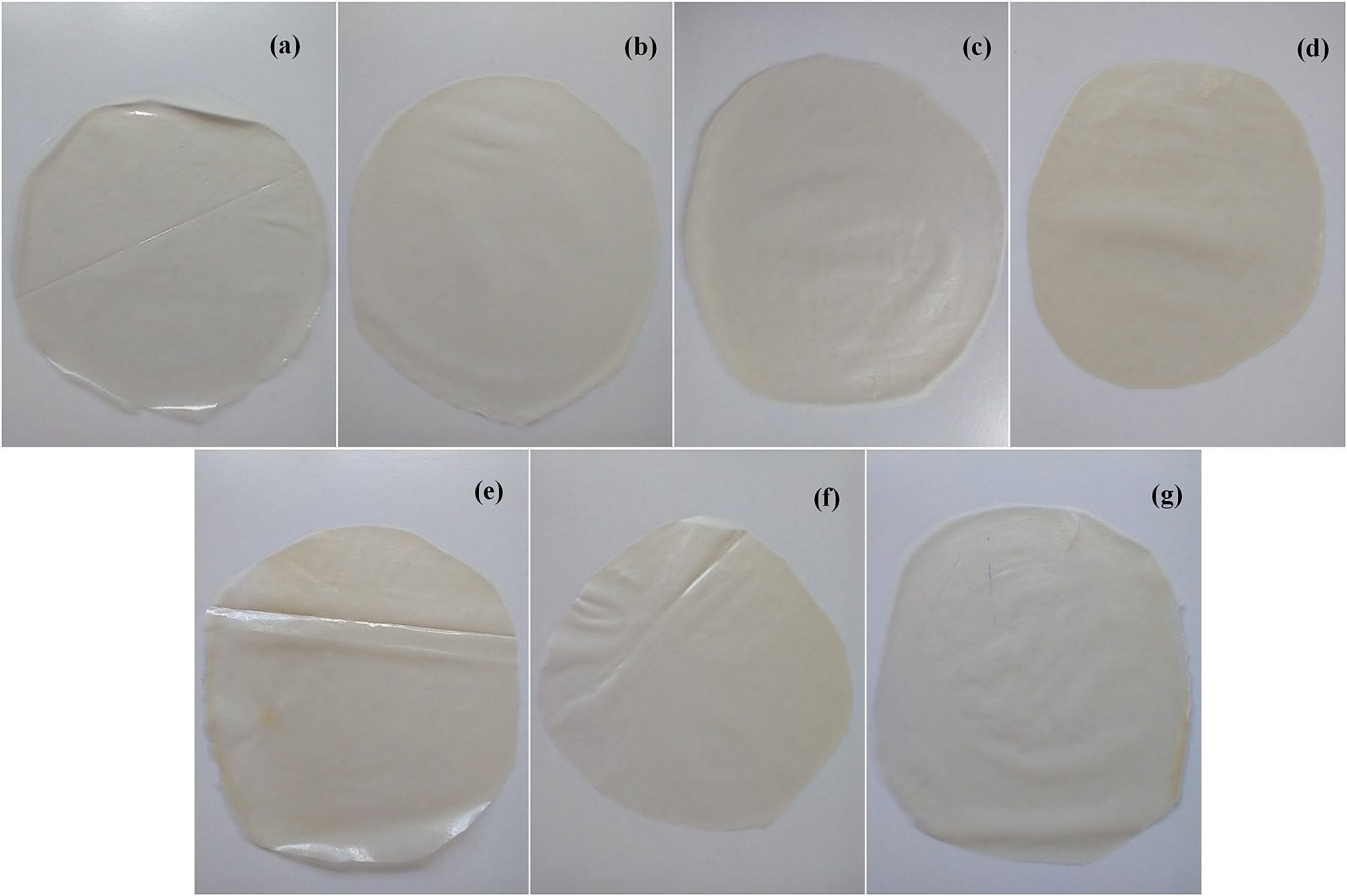
Biodegradable films: (**a**) FG; (**b**) 90FG/10CMC; (**c**) 80FG/20CMC; (**d**) 70FG/30CMC; (**e**) 90FG/10PVOH; (**f**) 80FG/20PVOH, (**g**) 70FG/30PVOH.

The biodegradable films produced, including FG and the FG/CMC and FG/PVOH mixtures can be observed in Fig. 3. The influence of CMC and PVOH concentrations added to filmogenic solutions was observed in the L^*^ parameter of the films, with a significant decrease. This behavior can be explained by the compaction of the filmogenic matrix, due to the reduction of the spacing between the polymer chains, reducing the passage of light through the film^56^, confirmed by the opacity results, which showed less transparency with the addition of polymers. Result similar to that obtained by^57^ in gelatin films with increasing concentrations of agar. The transparency values observed in this study in all composite films are lower than those reported for low density polyethylene (4.26 A600/mm), which is a widely commercialized synthetic plastic^58^.

### Fourier transform infrared spectroscopy (FTIR)

**Figure 4:**
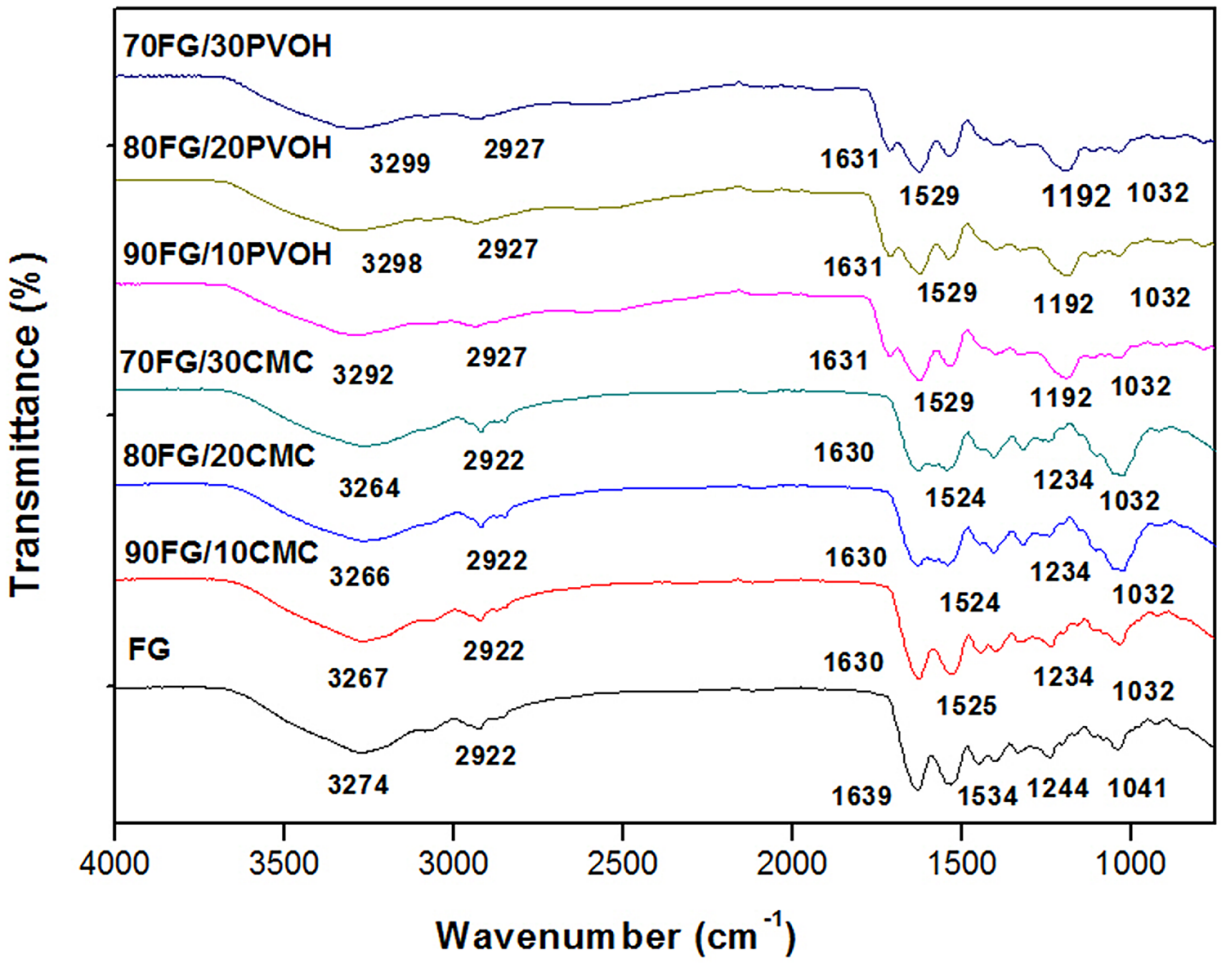
FT-IR spectra of FG film and FG/CMC and FG/PVOH mixtures.

The FTIR spectra of the FG control films and the FG/CMC and FG/PVOH compounds are shown in Fig. 4. The FG spectrum showed characteristic bands of gelatin in approximately 3274 cm^−1^ (amide A, representative of the elongation of the NH– bond coupled to the hydrogen bond); 2922 cm^−1^ (due to the elongation of H); 1639 cm^−1^ (amide I, representative of C=O, hydrogen bond coupled to COO); 1534 cm^−1^ (amide II, representative of the flexion of NH groups coupled to CN elongation) and 1244 cm^−1^ (amide III, representative of the vibrations in the CN and NH bond plane of vibrations of the bound amide or groups of glycine CH_2_)^59,60^. The band located at 1041 cm^−1^ also corresponds to glycerol (group –OH) added as a plasticizer.

Analyzing the FG/CMC spectra, it was possible to observe that after the addition of CMC, some peaks were shifted to new frequencies. For example, the amide I peak was shifted from 1639 (FG film) to 1630 cm^−1^ in composite films in all studied proportions. Amide I is normally used to analyze the secondary structure of proteins^61^. The decrease in the wave numbers of this band may suggest the occurrence of structural changes in protein chains, such as in the helical structure of gelatin^62^. Meanwhile, the peak of amide II was changed from 1534 to 1525 cm^−1^ (90FG/10CMC) and 1524 cm^−1^ (80FG/20CMC; 70FG/30CMC), and that of 1244 amide III to 1234 cm^−1^. The changes in the spectra suggested the presence of protein-polysaccharide interactions via hydrogen bonding^49^. In addition, the peak 3274 cm^−1^, corresponding to the band amide A, was displaced by 3267 cm^−1^, due to the occurrence of hydrogen bonds between gelatin and CMC. The new intermolecular changes formed can result in a denser structure in the films^33^.

Regarding the FG/PVOH film, variations in the absorption in the region of amides I, II and III were observed when PVOH was incorporated in different proportions in the gelatin, indicating the establishment of chemical bonds. It was observed that in these films the peak referring to amide I, located at 1639 cm^−1^ was slightly shifted to 1631 cm^−1^, regardless of the polymer concentration. The band amide I is the most sensitive of the gelatin, and such behavior suggests the occurrence of interactions through hydrogen bonds between the gelatin carbonyl group and the PVOH hydroxyl group, increasing intermolecular forces, which may, in a way, indicate the occurrence of crystallinity in the films produced^63^. The presence of PVOH also influenced the amide II band, causing a displacement from 1534 to 1529 cm^−1^, as well as affecting the characteristic signs of the amide III band, which was changed from 1242 to 1192 cm^−1^. It was also identified the change in the peaks related to amide A, which were moved to higher frequencies with the increase in the proportion of PVOH. For these mixtures, the peaks located in the 3500–3200 cm^−1^ region refer to the stretching vibration of the inter and intramolecular hydrogen bonds, which indicates the presence of hydrogen bonds between gelatin and PVOH.

### Thermogravimetric analysis (TGA)

**Figure 5:**
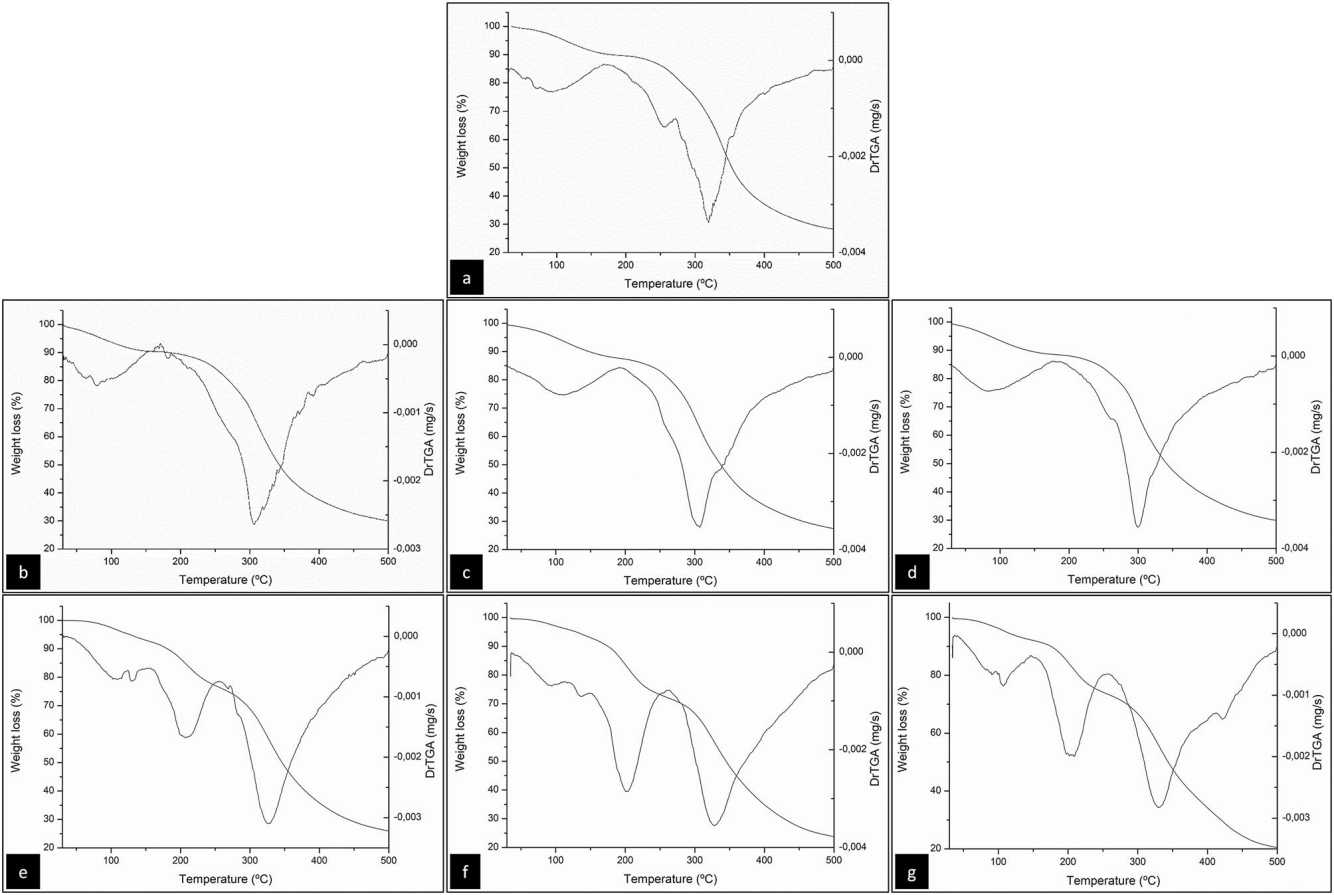
TGA and DTGA thermograms of the control film (FG) and mixing films: (**a**) FG; (**b**) 90FG/10CMC; (**c**) 80FG/20CMC; (**d**) 70FG/30CMC; (**e**) 90FG/10PVOH; (**f**) 80FG/20PVOH; (**g**) 70FG/30PVOH.

The TGA analysis evaluated the effect of adding CMC and PVOH on the thermal stability of the gelatin-based films and the TGA and DTGA curves are shown in Fig. 5. The FG and FG/PVOH films showed three stages of weight loss, while the FG/CMC mixtures showed only two stages. In the first stage, all films showed a loss of mass of 6–8.5% in the temperature range of 30–130 °C. This stage is associated with the loss of free and absorbed water^64^.

In the control film (FG), the second stage occurred between 225 and 265 °C, with a loss of mass around 5%, probably associated with low molecular weight protein fractions and the plasticizer^65^. And in the third stage, a loss of mass of about 34% was observed at 275–345 °C, being attributed to the thermal degradation of peptide bonds in the main chain of gelatin^66^.

FG/CMC films, in the second stage, showed weight loss of approximately 37% at 240–345 °C, probably due to protein and polysaccharide degradation. Therefore, it is possible to notice that the addition of CMC did not influence the thermal resistance of the films, which remained stable compared to the pure gelatin films.

The FG/PVOH films, in the second stage, showed mass loss of 14–16% at 180–250 °C, while the third stage was observed at 275–360 °C with weight loss between 26 and 29%. The reduction in the initial degradation temperature of these films compared to FG/CMC reveals that the molecular interactions between gelatin and PVOH affected the thermal behavior of the films, indicated by the decrease in thermal stability.

### X-ray diffraction

**Figure 6:**
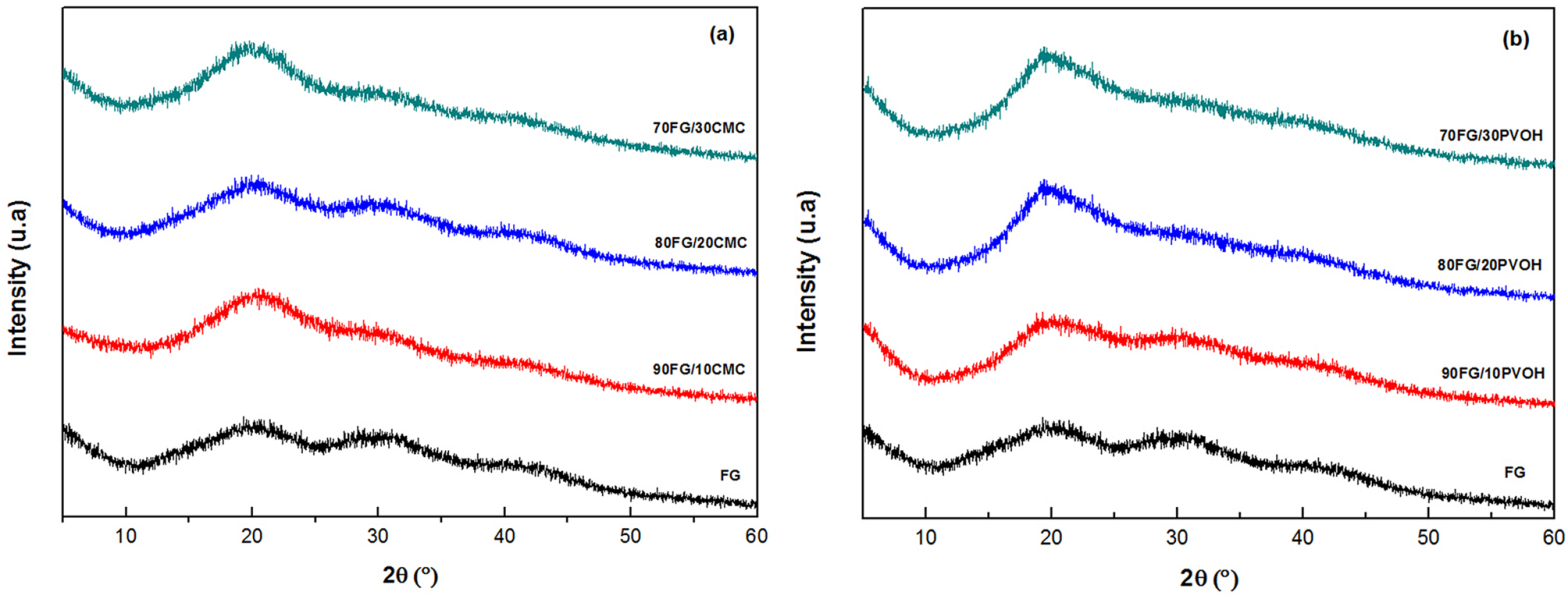
(**a**) XRD pattern of FG/CMC and FG films and (**b**) FG/PVOH and FG films.

XRD analysis was performed to check for a possible change in crystallinity of gelatin films when adding other polymers to this matrix (Fig. 6). The XRD pattern of the FG film showed two diffraction peaks, at 2θ = 7°–8°, attributed to the presence of a small amount of triple helical structure, characteristic of collagen, accompanied by the wide diffraction peak at 2θ = 21° which corresponds to the amorphous halo of the protein^67^.

It can be seen in Fig. 6a that all FG/CMC films presented XRD profiles similar to FG, however, with greater intensity, indicating that CMC was well dispersed in the gelatin matrix. The increase in peak intensity was the result of interactions between polymers, through intermolecular hydrogen bonds between gelatin and CMC, producing more ordered structures, indicating an increase in crystallinity in the film matrix. Increase in peak intensities was also observed by^33^ in gelatin films and epigallocatechin gallate.

The diffractograms shown in Fig. 6b demonstrated that the different proportions of PVOH produced films with typical characteristics of semicrystalline material. In porcine gelatin and PVOH films, other authors^68^ obtained a semicrystalline diffraction peak around at a 2θ = 20°, similar to what was observed in this work. Similar patterns of XRD, too, have been observed in studies by^69^ in pure PVOH films. According to^42^, PVOH has a flexible structure, which allows the packaging of molecules and crystallization, while gelatin crystallizes due to its tendency to resaturation^61^.

### Scanning electron microscopy (SEM)

**Figure 7:**
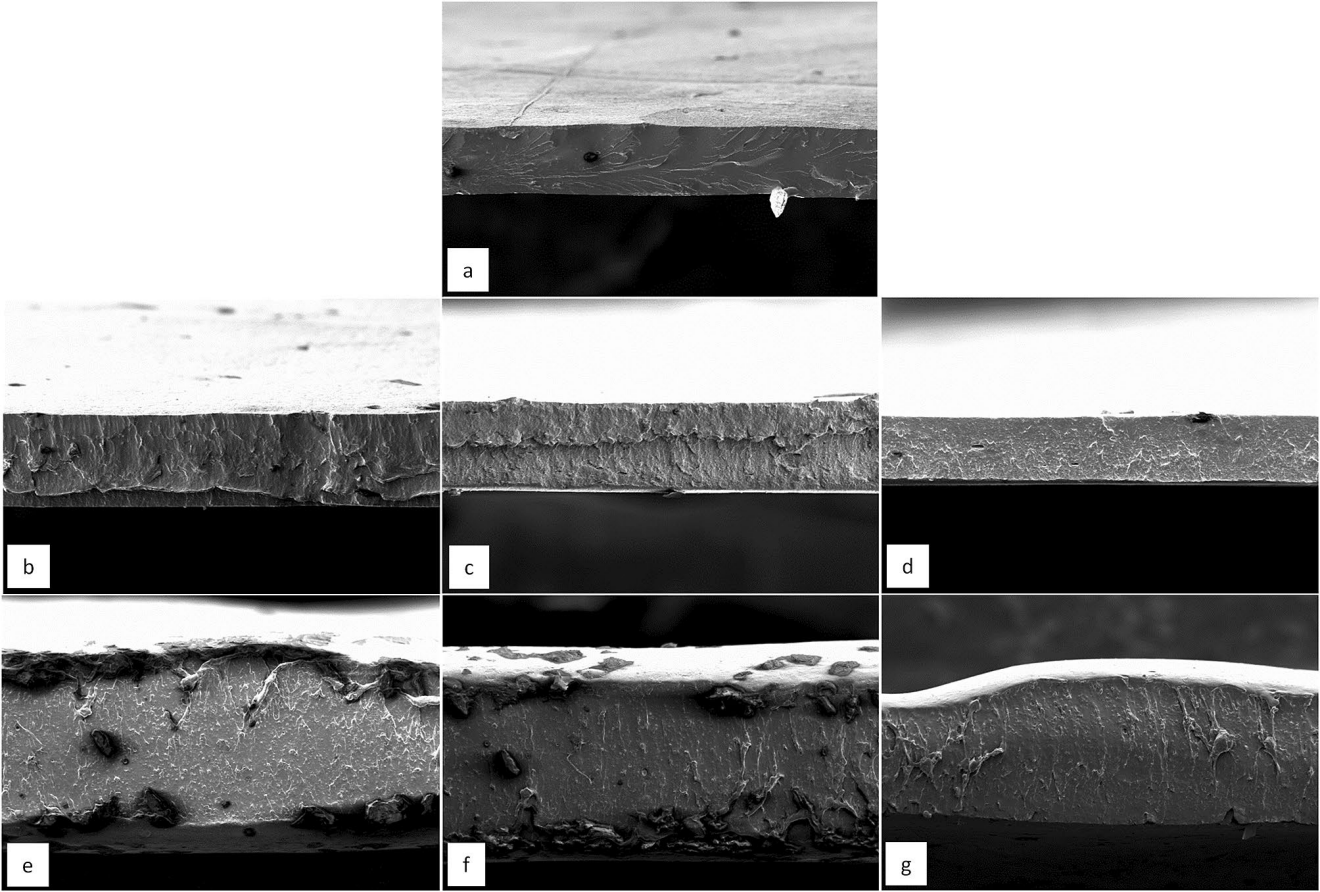
Cross-sectional images of the FG film and FG/CMC, FG/PVOH mixtures in different proportions.

The microstructure of the films was evaluated to verify the influence of the addition of CMC and PVOH on the structure of the films (Fig. 7). The transversal images of the FG film have a more homogeneous structure, while FG/CMC showed imperfections, which may be due to the presence of different macromolecules in the polymeric matrix and to the interactions between these components^70^. It is observed that the microstructures of the composite films FG/CMC and FG/PVOH are more uniform as the concentration of polymers increases, producing more compact and dense structures, which may have better mechanical and barrier properties. This structure may be due to intermolecular polymer associations or compatibility between components^71^.

The 70FG/30CMC mixture was identified as the most homogeneous of the studied proportions, confirmed by the greater mechanical resistance found for this composition. On the other hand, the presence of some roughness was more noticeable in the structure of the films with PVOH, being in accordance with the mechanical results, which indicated higher values of tensile strength for FG/CMC. However, no evidence of phase separation was observed in any of the composite films, demonstrating the compatibility of both polymer mixtures.

## Conclusion

The physical, chemical and functional properties of FG films were modified by the addition of CMC and PVOH. It was found that the incorporation of 10% of CMC or PVOH was sufficient to produce films with better mechanical resistance, water vapor barrier and water solubility properties, in relation to gelatin film.

FG/CMC films showed greater mechanical resistance, but were more sensitive to water than FG/PVOH. The SEM analysis suggested compatibility between the components of the films. The 70FG/30CMC film showed greater tensile strength, while the 70FG/30PVOH obtained less solubility in water.

The results indicated that the polymeric mixtures studied in this work are adequate to improve the functional properties of fish gelatin films. What can expand the possibilities of making films with potential application in the packaging sector, with the advantage of biodegradability and low production cost, with the use of waste and the consequent reduction of environmental impact.

## Supplementary Information

Supplementary Information.

## Data Availability

The datasets generated and/or analyzed during the current study are available from the corresponding author on reasonable request.
