# Supplementary material for: Effect of polyvinyl alcohol and carboxymethylcellulose on the technological properties of fish gelatin films
Source: Sci Rep. 2022 Jun 21;12:10497. doi: 10.1038/s41598-022-14258-y (PMC9213542; doi:10.1038/s41598-022-14258-y)
Supplement: Supplementary file 1 — Supplementary Information. [file 41598_2022_14258_MOESM1_ESM.docx]

**Effect of polyvinyl alcohol and carboxymethylcellulose on the technological properties of fish gelatin films**

#### Gleyca de Jesus Costa Fernandes^1*^, Pedro Henrique Campelo^2^, Jayne de Abreu Figueiredo^3^, Hugo Junior Barbosa de Souza^3^, Maria Regina Sarkis Peixoto Joele^4^, Maria Irene Yoshida^5^, Lúcia de Fátima Henriques Lourenço^1^

^1^Animal Research Laboratory - LAPOA, Graduate Program in Food Science and Technology - PPGCTA, Federal University of Pará - UFPA, Belém, PA, Brazil.

^2^Department of Food Technology, Federal University of Vicosa, Av. PH Rolfs, s/n, Vicosa, MG 36570-900, Brasil.

^3^Food Science Department, Federal University of Lavras, 37200-000, Lavras, MG, Brazil.

^4^Pará Federal Institute of Education, Science and Technology - IFPA, Castanhal, PA, Brazil.

^5^Chemical Department, Federal University of Minas Gerais, Belo Horizonte/ MG, Brasil.

*Corresponding author: gleycafernandes@yahoo.com.br. Phone: +55 35 985670393 ORCID: https://orcid.org/0000-0002-5364-4745

**Appendix A. Supplementary data**

**Table A. 1** Average values and standard deviations of thickness, tensile strength (TS), elongation (% E), water vapor permeability (WVP) and solubility of the films.

| **Movies** | **Thickness**  **(mm)** | **TS**  **(Mpa)** | **Elongation**  **(%)** | **WVP**  **(x10^-11^ gmm^-2^.s^-1^.Pa^-1^)** | **Solubility**  **(%)** |
| --- | --- | --- | --- | --- | --- |
| **FG** | 0.078 ± 0.01^c^ | 4.23 ± 0.96^g^ | 115.70 ± 1.71^d^ | 7.33 ± 0.11^a^ | 63.35 ± 0.92^a^ |
| **FG/CMC** |  | | | | |
| 90/10 | 0.105 ± 0.02^b^ | 21.33 ± 0.93^b^ | 107.94 ± 1.85^e^ | 5.32 ± 0.11^b^ | 48.01 ± 1.03^b^ |
| 80/20 | 0.111 ± 0.04^b^ | 28.95 ± 1.19^c^ | 104.09 ± 1.99^f^ | 5.53 ± 0.13^b^ | 37.83 ± 0.08^c^ |
| 70/30 | 0.114 ± 0.02^b^ | 36.58 ± 0.93^a^ | 101.59 ± 1.23^g^ | 5.84 ± 0.23^b^ | 21.74 ± 0.73^d^ |
| **FG/PVOH** |  |  |  |  |  |
| 90/10 | 0.132 ± 0.04^a^ | 6.78 ± 0.51^f^ | 345.03 ± 1.61^a^ | 5.38 ± 0.02^b^ | 20.15 ± 1.24^d^ |
| 80/20 | 0.161 ± 0.04^a^ | 9.11 ± 0.44^e^ | 291.39 ± 1.28^b^ | 5.87 ± 0.19^b^ | 13.55 ± 1.64^e^ |
| 70/30 | 0.182 ± 0.03^a^ | 13.41 ± 0.83^d^ | 223.77 ± 1.66^c^ | 6.01 ± 0.75^b^ | 8.52 ± 0.89^f^ |

Different letters in the same column indicate a significant difference (p <0.05).

**Table A. 2** Average values and standard deviations of color parameters (CIELAB) and film opacity.

| **Movies** | **Color parameters** | | | | **Opacity** |
| --- | --- | --- | --- | --- | --- |
|  | **L *** | **a *** | **b *** | ***△* E** |  |
| **FG** | 93.35 ± 0.51^a^ | -0.85 ± 0.03^b^ | 8.15 ± 0.25^b^ | - | 2.02 ± 0.01^g^ |
| **FG/CMC** |  |  |  |  |  |
| 90/10 | 92.63 ± 0.07^b^ | -0.98 ± 0.02^c^ | 11.22 ± 0.67^a^ | 3.47 ± 0.45^a^ | 2.36 ± 0.01^e^ |
| 80/20 | 92.01 ± 0.52^c^ | -1.07 ± 0.03^d^ | 11.76 ± 0.13^a^ | 3.71 ± 0.24^a^ | 2.91 ± 0.01^b^ |
| 70/30 | 91.27 ± 0.19^d^ | -1.13 ± 0.02^d^ | 11.30 ± 0.80^a^ | 3.80 ± 1.03^a^ | 3.07 ± 0.01^a^ |
| **FG/PVOH** |  |  |  |  |  |
| 90/10 | 90.75 ± 0.57^d^ | -0.51 ± 0.03^a^ | 10.71 ± 1.67^a^ | 3.75 ± 1.34^a^ | 2.33 ± 0.01^f^ |
| 80/20 | 89.55 ± 1.01^d^ | -0.48 ± 0.01^a^ | 10.65 ± 0.97^a^ | 4.72 ± 0.59^a^ | 2.73 ± 0.01^d^ |
| 70/30 | 89.29 ± 0.68^d^ | -0.46 ± 0.01^a^ | 11.08 ± 0.01^a^ | 5.02 ± 0.72^a^ | 2.89 ± 0.01^c^ |

Different letters in the same column indicate a significant difference (p < 0.05).
